# The implementation and public health impacts of cannabis legalization in Canada: a systematic review

**DOI:** 10.1111/add.16274

**Published:** 2023-06-28

**Authors:** Wayne Hall, Daniel Stjepanović, Danielle Dawson, Janni Leung

**Affiliations:** ^1^ National Centre for Youth Substance Use Research The University of Queensland Brisbane Australia

**Keywords:** cannabis economics, cannabis policing, cannabis potency, cannabis use disorders, hyperemesis disorders, psychoses

## Abstract

**Aims:**

We provide a narrative summary of research on changes in cannabis arrests, cannabis products and prices, cannabis use and cannabis‐related harm since legalization.

**Methods:**

We systematically searched for research on the impacts of cannabis legalization in Canada in PubMed, Embase, Statistics Canada and government websites and Google Scholar, published between 2006 and 2021.

**Results:**

Cannabis legalization in Canada has been followed by substantial reductions in cannabis‐related arrests and cannabis prices. It has also increased adults’ access to a diverse range of cannabis products, including edibles and extracts. The prevalence of cannabis use among young adults has increased, but there have been no marked increases or decreases in use among high school students or changes in the prevalence of daily or near‐daily use. Legalization has been associated with increased adult hospital attendances for psychiatric distress and vomiting, unintentional ingestion of edible cannabis products by children and hospitalizations for cannabis use disorders in adults. There is conflicting evidence on whether cannabis‐impaired driving has increased since legalization. There is suggestive evidence that presentations to emergency departments with psychoses and cannabis use disorders may have increased since legalization.

**Conclusions:**

Legalization of cannabis in Canada appears to have reduced cannabis arrests and increased access to a variety of more potent cannabis products at lower prices. Since 2019, recent cannabis use in Canada has modestly increased among adults but not among adolescents. There is evidence of increased acute adverse effects of cannabis among adults and children.

## INTRODUCTION

In October 2018, Canada became the second nation after Uruguay to legalize the production and sale of cannabis for adult use. Medical cannabis use was legalized in Canada in 2001, and the liberalization of regulations over the next two decades facilitated patient access [1].

During the 2015 Canadian Federal election campaign, the Liberal Party promised to ‘legalize, regulate, and restrict access to marijuana’ [2]. The party argued that cannabis legalization would ‘ensure that we keep marijuana out of the hands of children, and the profits out of the hands of criminals’ and ‘regulate and restrict access to marijuana’ (cited in [3]). The Cannabis Act (Bill C‐45; see [3, 4]) was passed in June 2017 and enacted in October 2017. It was implemented in October 2018 to give provincial and territorial governments a year to design cannabis retail systems and pass necessary legislation [1, 3]. The Cannabis Act [4] had seven priorities (see Box 1).

Box 1 **Priorities of the Cannabis Act**

The priorities of the Cannabis Act (Minister of Justice 2015)

- Protect the health of young persons by restricting their access to cannabis;
- minimize inducements to use cannabis;
- allow the legal production of cannabis to replace the illicit cannabis market;
- deter illicit cannabis production and sale by appropriate sanctions and enforcement;
- reduce the burden that dealing with cannabis offences imposed on the criminal justice system;
- enable cannabis users to have a quality‐controlled supply of cannabis; and
- increase public awareness of the health risks of using cannabis.

A separate bill (C‐46) defined cannabis‐impaired driving [5]. On expert advice [6], the bill defined a level of two nanograms of tetrahydrocannabinol (THC) per millilitre of blood as *per se* evidence that a driver was cannabis‐impaired. Defining cannabis‐related impairment in terms of a THC *per se* blood limit was challenging, because many other factors influence the level of impairment [7]. The government delayed the sale of cannabis extracts and edibles until October 2019 to allow for more deliberation on how to regulate them [8]. The act limited the THC dose of edibles to 10 mg but did not otherwise limit the THC levels in cannabis products [9].

### The implementation of cannabis legalization in Canada

#### Federal law and regulation

In the Canadian federal system, the federal government enacts legislation on criminal offences that are enforced by the provinces [8]. Cannabis legalization accordingly required federal legislation to remove criminal penalties for adult cannabis use and to license cannabis producers and wholesalers. This legislation also set a minimum legal purchase age for cannabis, required relatively plain packaging and warning labels on cannabis products and restricted the promotion of cannabis products [10]. The Cannabis Act allowed individuals aged more than 18 years to purchase herbal cannabis or cannabis oil from a licensed retailer or on‐line store. Adults could possess up to 30 g of (dried) cannabis in public, and they could share this amount of cannabis with other adults. They could also grow up to four cannabis plants per residence for their personal use or to share with friends.

The federal government collects taxes on licensed cannabis products and shares the revenue with provincial and territorial governments. Cannabis was taxed at $1 Canadian per gram (US$0.77) or 10% of the sales price, whichever was the higher [10]. The federal government retains 25% of the revenue and passes the remainder to provincial governments [8]. A 5% Goods and Services Tax is applied to all goods in Canada at the point of sale, and was also applied to cannabis. The provinces may also add a provincial sales tax (PST), which varies from zero in Alberta and the Northwest, Nunavut and Yukon Territories to 10% in some Atlantic provinces [8].

### Provincial and territorial roles

Provincial and territorial governments legislated to regulate cannabis production, distribution and sales and set limits on the amount of cannabis an adult could possess. The regulations of retail sales were loosely modelled on the provinces’ approaches to regulating alcohol sales [10]. Provinces with an alcohol monopoly (e.g. Québec) adopted a cannabis retail sales monopoly in which profits from sales were used to fund cannabis regulation and prevention and treatment services. Alberta, which allows commercial alcohol retail sales, adopted the same approach to cannabis retail sales. Other provinces adopted a mixed retail model that allowed for‐profit retail cannabis sales from storefronts and mail purchases from government online and retail stores (see Supporting information, Table S1).

The minimum legal age for cannabis purchase varied between provinces from 18 to 21 (Supporting information, Table S1). Quebec set the minimum legal purchase age to 21 years, reduced the amount of cannabis that an adult could legally possess and banned the growing of cannabis for personal use. It also imposed limits on the THC content of cannabis edibles and extracts, setting a maximum of 5 mg THC per portion of edibles and a maximum THC content of 30% by weight in extracts [11]. It also banned additives to any cannabis products that would increase their attractiveness or flavour or enhance their psychoactive effects.

### Assessing the effects of cannabis legalization in Canada

Given the policy priorities of Canada's cannabis legalization [4] we assessed the effects of cannabis legalization in Canada, 4 years since Canada legalized the production and sale of cannabis for adult use in 2018. We systematically reviewed and summarized research and existing data on the changes in cannabis‐related arrests, cannabis markets, sales and products and patterns of cannabis use and cannabis‐related harm since legalization. We used the findings to make suggestions on how future evaluations of the effects of cannabis legalization in Canada can be strengthened.

## METHODS

### Design

We conducted a systematic review of the published and grey literature for studies of the impacts of cannabis legalization in Canada. The review was not registered.

#### Eligibility criteria

We included research on the extent to which the aims of Canadian policy have been realized, for example, by reducing arrests; eliminating the illicit cannabis market; protecting youth; and minimizing cannabis‐related harms. Based on the population, exposure, comparison, outcome (PECO) research question method, the studies met inclusion criteria if they were (1) general population studies; (2) examined the impacts of cannabis legalization; (3) compared the impacts from before legalization; and (4) reported on quantitative data on outcomes. Studies were excluded if they were from non‐representative samples (e.g. patient groups, specific ethnic groups, high‐risk subpopulations) or did not report quantitative data on the impacts of cannabis legalization in Canada.

### Search and selection process

We systematically searched for evidence in PubMed and Embase. We also conducted supplementary hand searches on Government of Canada's cannabis research and data website, Statistics Canada's health statistics and reports, Google and Google Scholar.

We searched for records with terms related to cannabis legalization and the outcomes in the titles or abstracts or MeSH terms (see Supporting information, S3 for search string). The search concept involved terms related to [(cannabis legalization) and (cannabis or other substance use, cannabis‐impaired driving or motor vehicle accidents, health service or mental health presentations)], with no restrictions on language or publication dates. The search was conducted on 7 June 2021 and updated on 29 August 2022, approximately 4 years since cannabis was first legalized in Canada in 2018. Screening and selection were carried out by two researchers, involving one reviewer for title screen and two reviewers in parallel for full‐text review.

### Synthesis methods

For each included study, we recorded the study aims, design and setting, year of data collected, outcome measures, effect sizes, the impact and key results and conclusions. All data were extracted by one reviewer and double‐checked by a second reviewer. Effect sizes, confidence intervals and significant testing results from each of the studies were extracted and tabulated. The Joanna Briggs Institute (JBI) critical appraisal tool was used to assess methodological quality of the included studies (Supporting information, S7).

Meta‐analyses were not conducted because of the large variations in the outcomes and time‐periods between the studies. Findings were summarized narratively. We looked for (1) evidence of an association between cannabis legalization and a change in a health outcome and (2) evidence that would exclude alternative explanations of an association; for example, increased attention by clinicians to cannabis use; or secular trends in use; etc. We report when there is evidence of an association, no association and when the evidence of an association is mixed. We also list plausible alternative explanations of associations between legalization and outcomes.

## RESULTS

### Study characteristics

Our search identified 1964 records, 1715 from the database search and 249 from supplementary searches. A total of 1808 unique titles were screened, and 167 reports were retrieved for full‐text screening. After screening, 19 studies were included in our review [see Supporting information, S4 for the Preferred Reporting Items for Systematic reviews and Meta‐Analyses (PRISMA) flow‐chart].

Studies of the impacts of cannabis legalization in Canada have used market data, population surveys and administrative data, such as police, trauma clinics, emergency department (ED) and hospital records.

The Canadian population surveys that have been analysed in the context of cannabis legalization include the National Cannabis Survey (NCS; ages 15+), Canadian Cannabis Survey (CCS; ages 16+); Canadian Student Tobacco, Alcohol and Drugs Survey (CSTADS; ages 12–18) and the International Cannabis Policy Study (ICPS; aged 16–65; see Supporting information, Table S2).

All studies scored 6–8 on the JBI tool (see Supporting information, S7). The most common limitation was the lack of control for confounding factors. In most cases, the authors were unable to adjust for other potential factors that may have explained the changes observed post‐legalization. For example, increased cannabis‐related presentations may have been associated with increased testing and reporting [12, 13]. The same may have been true of studies of THC detected when driving because of increased testing rates post‐legalization and because THC measured in blood or urine may not necessarily cause impairment.

A summary of the included evidence and findings on the impacts of cannabis legalization in Canada is presented in Table 1 (see Supporting information, S8 for detailed summaries of individual studies).

**TABLE 1:** Summary of evidence on the impacts of cannabis legalization in Canada.

Policy outcome	Change observed	Is change associated with legalization?	Is a causal relationship plausible?
Cannabis‐related arrests	A substantial reduction	Yes	Yes. This is an intended effect of reform because cannabis use and possession no longer offences. No changes in other drug offences
Potency of cannabis products	A substantial increase	Yes	Yes. Increases in potency were much larger than in countries where cannabis was illegal
Legal cannabis prices	A substantial decrease	Yes	Yes. An expected effect of legalizing the production and sale of a formerly illicit commodity. Similar to trends in prices in US states where cannabis is now legal
Adult cannabis use	A modest increase in past 3 months	Yes	Yes. An expected effect of legalizing an illegal commodity. Possibly affected by increased preparedness to report use after legalization
Cannabis use in adolescents	Mixed findings on recent use	No	Uncertain. Low statistical power in most surveys
Daily cannabis use	No clear findings	No	Uncertain. Low statistical power in most surveys
Cannabis impaired car crashes	Mixed findings from surveys and toxicology	Unclear	Uncertain. Difficult to define cannabis‐related impairment using blood THC
Acute cannabis‐related ED attendances adults	Substantial increase	Yes	Yes. Largest increases seen after expansion of retail sales. Similar changes in US states when cannabis sales became legal
ED attendances by children	Substantial increase from low baseline	Yes	Yes. Largest increases occurred after legal sales of cannabis edibles. Similar trends in other jurisdictions that have legalized edibles
Adult psychiatric presentations	Mixed evidence of increases for psychoses and CUDs	In larger provinces	Uncertain. Could reflect increased ascertainment of cannabis use since legalization

*Note*: Changes observed and the estimates from the individual studies that inform this summary table are available in Supporting information, S8.

Abbreviations: THC = tetrahydrocannabinol; ED = emergency department; CUD = cannabis use disorder.

### Cannabis‐related arrests

An analysis of police data on cannabis‐related arrests between 1987 and 2019 [14] found a sharp decline in the number of people arrested for cannabis possession and use after the legalization of adult use (see Supporting information, Fig. S5). Rates of police‐reported cannabis‐related offences peaked in 2010 (more than 200 offences per 100 k population), reduced to fewer than 100 offences per 100 k in 2017, then further reduced to fewer than 50 offences per 100 k population in 2019. The decline in cannabis‐related arrests began several years before legalization, which may be because police gave a lower priority to these arrests after legislation to legalize adult use passed in 2017. These trends are plausibly causally related to legalization, which eliminated these offences for adults, and reductions in drug offences were confined to cannabis because there were no declines in arrest rates for other drug‐related offences.

### Cannabis prices and markets

We identified a review [12] and a Canadian government report [15] that examined cannabis prices and markets. Provincial data on the number of legal retail outlets that have opened and on the dollar value of cannabis sales indicate that the volume of legal cannabis sales steadily increased in all jurisdictions after legalization (see Supporting information, Fig. S6.1, data drawn from [16]).

Cannabis prices declined during the first 2 years after cannabis legalization in most jurisdictions (see Supporting information, Fig. S6.2, data drawn from [10]). The steepest declines were in Alberta, British Columbia, Manitoba, Ontario and Saskatchewan. Québec began selling cannabis in government stores at a much lower price than the other provinces, and its prices remained below those in other jurisdictions (see figure from Gibbs *et al*. [10]). The decline in cannabis prices is plausibly a result of legalization, which lowered production costs because it permitted larger scale, more efficient legal production of cannabis. These were passed on to consumers in the form of lower cannabis prices [10, 17].

For the first year after legalization, only cannabis flower and oil could be legally sold to Canadian adults. The sale of edible cannabis products and cannabis extracts increased after they were allowed in October 2019, and they accounted for an increasing proportion of total national sales (see Supporting information, Fig. S6.3, data drawn from [18]). Legalization has also increased the availability of higher potency cannabis extracts [15].

### Impact on the illicit cannabis market

In surveys, the proportion of cannabis users who reported purchasing from legal sources or growing their own cannabis has steadily increased since legalization [10]. According to one estimate based on sales data, legal sales have accounted for an increasing proportion of all purchases in the 3 years since legalization [19]. In the NCS, the percentage of users who reported obtaining their cannabis from illegal sources declined from 51% in the first quarter of 2018 to 35% in the first quarter of 2020 [12], while the proportion who obtained cannabis from legal sources increased from 23 to 68% (see Supporting information, Fig. S6.4, data provided by Statistics Canada [18]).

### Patterns of cannabis use

A major difficulty in assessing the impact of legalization on cannabis use in Canada is the lack of regular population surveys of cannabis use in the two decades before legalization. The surveys conducted since 1984 used different questions to ask about cannabis use and did so over different time‐periods (e.g. the past year and the past 3 months) Two papers [12, 20] summarized data on patterns of cannabis use in these major population surveys.

Roterman & Macdonald [21] analysed survey data between 1985 and 2015 and concluded that the prevalence of cannabis use in the past year increased from 6 to 14% between 1985 and 2004. There were conflicting survey findings on whether cannabis use has increased since the early 2000s. The Canadian Community Health Survey—Mental Health and Well‐being survey found no change between 2002 and 2012 (12.2%), and the Canadian Alcohol and Drug Use Monitoring Survey reported no increase in past‐year cannabis use between 2008 and 2012 (11–10%). However, cannabis use in the past year increased in the Canadian Tobacco Use Monitoring Survey (CTUMS) and Canadian Tobacco, Alcohol and Drugs Survey (CTADS) from 9% in 2004 to 12% in 2015 [22].

There was more consistent survey evidence that the prevalence of past‐year cannabis use has increased since legalization among Canadian adults over the age of 25 years [10, 12, 16]. The surveys differed in whether the prevalence of cannabis use has changed among people under the minimum legal purchase age since legalization [23].

**FIGURE 1:**
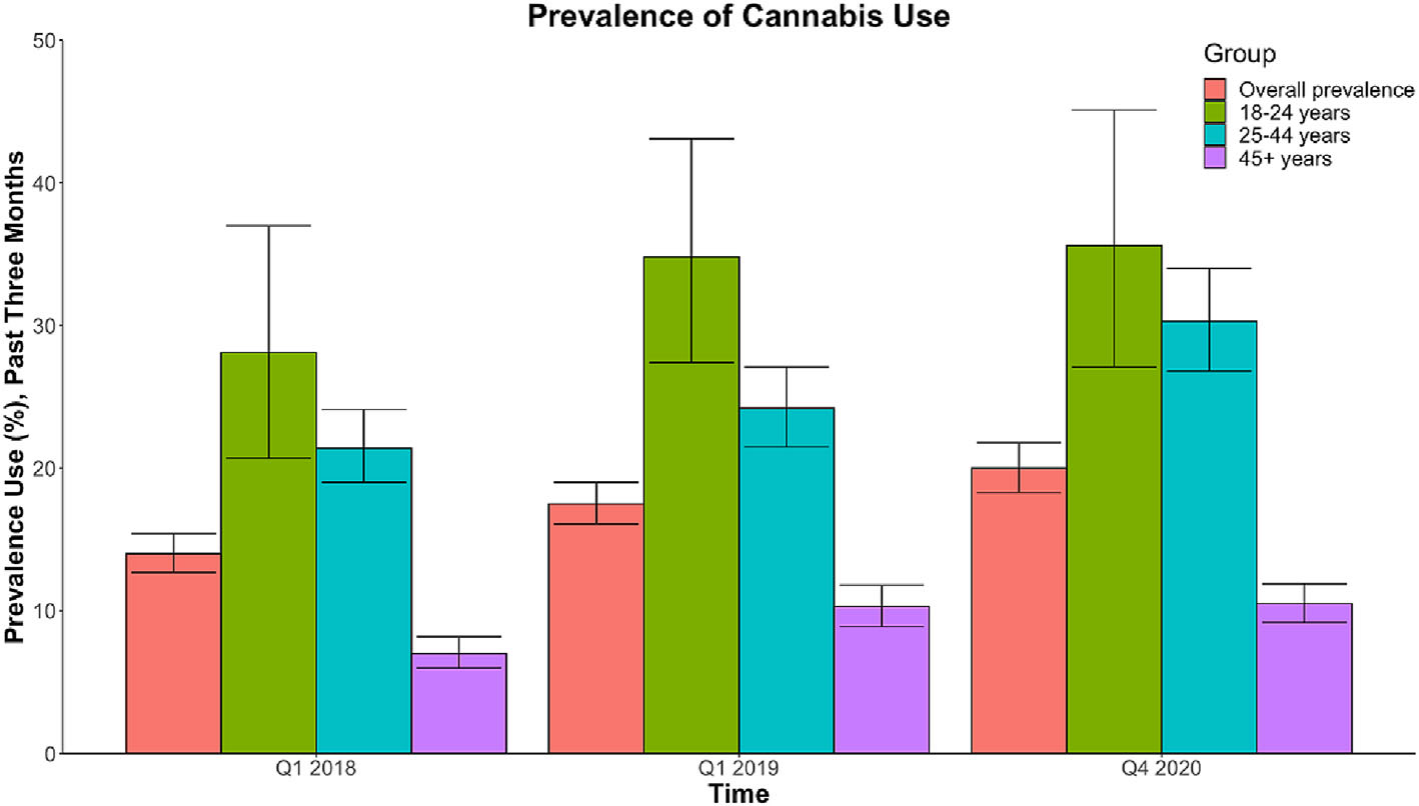
Prevalence of cannabis use in the past 3 months by time, relative to legalization (Q1 2018). Data drawn from the National Cannabis Survey (NCS) and provided by Statistics Canada [16].

In the NCS, the prevalence of cannabis use in the past 3 months increased from 14% in 2018 to 18% in 2019 and 20% in 2020 (see Fig. 1). Cannabis use was highest in those aged 18–24 years (28, 35 and 36%, respectively). In those aged 25–44 years, the prevalence in 2020 (30%) was significantly higher than in 2018 (21%) and 2019 (24%). In the 45+ group, there was a significant increase from 2018 (7%) to 2019 (10%) and 2020 (11%). The estimated prevalence of use among people under the legal age to purchase cannabis [15, 16, 17] fluctuated (20% in 2018, 10% in 2019 and 19% in 2020) but these estimates did not differ significantly, possibly because of the small sample sizes in these age groups.

In the CCS, the percentage of adults who reported cannabis use in the past 12 months increased from 22% in 2018 to 25% in 2019, 27% in 2020 and 25% in 2021 [23]. Cannabis use in the past year increased in those aged 20–24 years (44, 51, 52 and 49%, respectively) and 16–19 years (37, 44, 44 and 38%, respectively) [23].

By contrast, there were no substantial increases or decreases seen in cannabis use among adolescents. The CSTADS found that cannabis use in the past year was steady among those under the legal purchase age: 17% in 2016/2017 and 18% in 2018/2019. Curiously, there was a statistically significant increase in cannabis use among students aged 13–15 from 6% in 2016/2017 to 7% in 2018/2019, but there was not a significant difference in the prevalence of cannabis use among students aged 15–17 (28% in 2016/2017 and 29% in 2018/2019) [12].

Pham *et al*. [20] examined changes in cannabis use after legalization using data from the Canadian Tobacco and Nicotine Survey. The 2017 CTADS provided the baseline estimate of cannabis use prevalence (*n*  = 16 349), and the 2019 Canadian Tobacco and Nicotine Survey measured prevalence after legalization (*n*  = 8614). Unfortunately, the items inquiring about cannabis use in each survey differed in their wording. Pham *et al*. approximated a measure of cannabis use in the past 30 days on which cannabis use increased from 9% in 2017 to nearly 11% [95% confidence interval (CI) = 10.1, 11.7] in 2019.

### Frequency of use

In the NCS, the prevalence of ‘daily and near‐daily’ use of cannabis (see Supporting information, Fig. S7; data drawn from [24]) did not change between 2018 and 2019 (6 and 6%) but marginally increased to 8% in 2020 [12, 16]. The percentage of cannabis users who were daily/near daily users did not significantly increase over the 3 years (40, 36 and 40%).

In the CCS, the proportion of daily or near daily users among those who used cannabis in the past 12 months was stable: 25% in 2018, 24% in 2019 and 25% in 2020. In 2020, the rate of daily or almost daily use was highest among males (29%) and people aged 25 + years (26%).

#### Route of administration

In the NCS, smoking was the most common way of using cannabis (65% in 2019 and 58% in 2020), followed by edible products (13 and 19%) and ‘vaping’ cannabis oils (13 and 12%). The most commonly used cannabis products were ‘dried flower or leaf’ (79% 2018; 78% in Q1/2019; 71% 2020), ‘edibles’ (32, 29 and 41%) and ‘cannabis vape pens’ (20, 18 and 23% [12]).

In the CCS, the prevalence of cannabis smoking decreased from 89% in 2018 to 84% in 2019, 79% in 2020 and 74% in 2021 [23]. The use of edible products increased from 43% in 2018 to 48% in 2019 and 53% in 2020. Vaping increased from 33% in 2018 to 36% in 2019 before decreasing to 31% in 2020, possibly in response to the outbreak of lung injuries in the United States linked to vaping adulterated cannabis oils [12, 25].

Among CSTADS grades 7–12 students, ‘smoking a joint’ was the most common method of use in the past year. This marginally decreased from 80% in 2016/17 to 76% in 2018/2019. The use of edibles increased from 34 to 45%, while vaping increased from 30 to 42% and ‘dabbing’ from 22 to 28% [12].

### Public health and safety after legalization

#### Driving after cannabis use

We summarized findings from three papers on driving after cannabis use [12, 26, 27]. Since cannabis was legalized, fewer Canadian cannabis users report driving when they may be intoxicated. In the NCS, the proportion of cannabis users who reported driving a vehicle within 2 hours in the past 3 months was 14% in 2018 and 13% in 2019. The CCS (which asked participants slightly different questions in the 2 years) reported that the proportion who drove after using cannabis decreased from 27% in 2018 to 24% in 2019 and 19% in 2020 [12]. In the Centre for Addiction and Mental Health (CAMH) Monitor, the percentage of cannabis users who reported driving within 1 h of cannabis use decreased from 13% in 2017 to 12% in 2019 [12].

**FIGURE 2:**
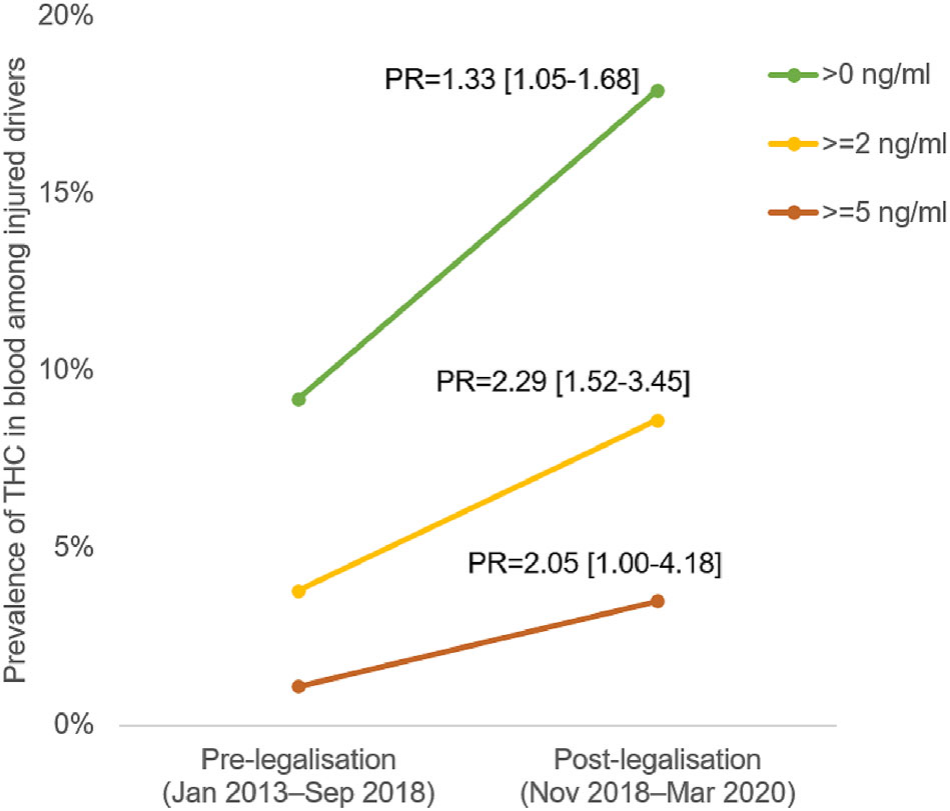
The prevalence of tetrahydrocannabinol (THC) in blood among injured drivers in British Columbia. PR = prevalence ratios of after versus before legalization from a log‐binomial regression model, adjusting for annual trend, season, sex, age group, health authority, injury severity and time and type of collision. Data drawn from Brubacher *et al.* [26].

The survey data appear to conflict with toxicological data from drug‐impaired driving cases and roadside surveys reported by Public Safety Canada (PSC [28]). These data show an increase in the proportion of cannabis‐impaired driving incidents (see Fig. 2). The PSC findings were supported by a recent study of THC in the blood of people who were injured in a car crash and hospitalized in four Vancouver hospitals between 2013 and 2020 [26]. The study reported the prevalence of three levels of THC in the blood in 3550 people before (January 2013 to September 2018) and 789 people after (November 2018 to March 2020) cannabis was legalized in 2018. The prevalence ratios of three levels of THC in blood increased by 1.33 (1.05–1.68) to 2.29 (1.52–3.45) times after 2018 [26]. There was no change in the proportion of injured people who were impaired by alcohol [prevalence ratio (PR) = 0.90 (0.71–1.14)]. By contrast, provincial ED records in Alberta and Ontario between 2015 and 2019 did not show any increase in weekly traffic‐injury presentations [27].

#### ED visits

Nine papers [29, 30, 31, 32, 33, 34, 35, 36, 37] reported on poison service calls and ED visits for cannabis‐related reasons, such as intoxication, unintentional ingestions and hyperemesis. Yeung *et al*. [33] analysed the proportion of ED visits that were cannabis‐related in Calgary and Edmonton in 11 770 visits before (2013–18) and 2962 visits after adult cannabis was legalized (2019). After legalization, there were small but statistically significant increases in cannabis‐related ED visits [incidence rate ratio (IRR) = 1.45]. There was also a significant increase in telephone calls to poison control hotlines regarding cannabis (IRR = 1.87). In an analysis of cannabis‐related paediatric ED visits in 2013–20 in urban Alberta there was a significant increase in hyperemesis presentations in 15–17‐year‐olds [rate ratio (RR) = 1.64], but not in younger age groups [34]. Paediatric visits for unintentional cannabis ingestion also increased in children aged 0–11 (RR = 1.24) and 15–17 (RR = 1.48) years.

Baraniecki *et al*. [30] compared cannabis‐related ED visits in St Joseph's Healthcare in Ontario in the 6 months before and after legalization in 2018–19. There was no statistically significant change in overall rates of ED visits for acute cannabis intoxication, but there was a 56% increase in presentations among people aged 18–29. The authors suggested that these trends may have reflected the increased the use of more potent cannabis products by new cannabis users and the increased questioning of patients by health workers regarding their cannabis use after legalization.

Auger *et al*. [29] investigated cannabis‐related hospitalizations among youth before and after the legalization of recreational cannabis in Quebec. In boys aged 10–14 years, cannabis‐related hospitalization rates increased from 5.2 per 100 000 1 year before legalization to 9.5 per 100 000 after legalization. Cannabis was mentioned in 39% of substance‐related hospitalizations among boys aged 10–14 years before legalization and in 70% after legalization.

Myran *et al*. [35] reported trends in ED visits attributable to cannabis use in the province of Ontario (population 13.8 million) between January 2016 and May 2021. They performed an interrupted time‐series analysis on the rates of ED visits during three phases in cannabis legalization in Ontario. The first phase was the pre‐legalization time period (January 2016 to September 2018). In the second phase there were strict limits on the number of licensed cannabis retail stores (October 2018 to February 2020). In the third phase—a fully commercialized phase—there were no limits on the number of retail cannabis outlets (March 2020 to May 2021). The third phase coincided with the COVID‐19 pandemic in Ontario.

The monthly rate of cannabis‐attributable ED visits was increasing before legalization, but there was a statistically significant 18% decline in the monthly rate 17 months after legalization with strict retail control (February 2020) Cannabis‐attributable ED visits decreased during the first 2 months of the COVID‐19 pandemic because of lower than usual ED use. When data for these 2 months were excluded, cannabis‐attributable ED visits significantly increased by 22% after commercialized retail sales compared to the period when strict retail store controls were in place. In all time‐periods, people aged 15–24 years had a higher rate of cannabis‐attributable visits than older age groups.

#### Paediatric cannabis‐related ED attendances

Myran *et al*. [36] investigated changes in the rate of accidental cannabis poisonings in Ontario among 2.35 million children aged 0–9 years between 1 January 2016 and 31 March 2021. The study included three periods: before legalization (January 2016 to September 2018); the period after only flower products were legalized (October 2018 to January 2020); and the period after which commercial edibles were sold (February 2020 to March 2021). There were 522 ED visits attributable to cannabis in children during the study: 81 before legalization, 124 visits in the first period of legalization and 317 visits in the second phase. The proportion of cannabis‐related ED visits that required hospitalization significantly increased after edibles could be legally sold (122 versus 29 in the first period and 20 before legalization). Nineteen ED visits (3.6%) required admission to an intensive care unit (ICU), but there were no deaths. Cannabis‐related ED visits increased from January 2016 to March 2021 in both the first (IRR = 3.13) and second (IRR = 9.12) periods. There was an increase in visits during the period when edibles could be sold compared to the preceding period when only flower could be sold (IRR = 2.23). This period coincided with the onset of the COVID‐19 pandemic, which was more likely to have reduced than increased ED visits.

Cohen *et al*. compared trends in ICU admissions for cannabis intoxication in children aged 0–18 years who were admitted to EDs between 2008 and 2019 before and after cannabis legalization [31]. There were 232 before and 66 after legalization in 2019. There were no significant increases in monthly cannabis‐related presentations (2.1 pre to 1.7 post), but there was a significant increase in the proportion admitted to the ICU (13.6 after versus 4.7% before legalization). There were more admissions of children under 12 years of age with after the ingestion of edible cannabis products.

Coret & Rowan‐Legg [32] examined the frequency of paediatric ED visits for cannabis intoxication before and after legalization at the Children's Hospital of Eastern Ontario between March 2013 and September 2020. They identified 37 patients (22 male), mean age 5.9 ± 3.8 years, 32 of whom (86%) were admitted in the 2 years after legalization. The most common symptoms were altered levels of consciousness, lethargy or somnolence, tachycardia and vomiting. Edibles were involved in 76% of cases. A third (32%) of cases required admission to the hospital for fewer than 24 hours.

Myran *et al*. [38] compared trends in paediatric hospitalizations for cannabis intoxication in the four Canadian provinces that included 86% of the Canadian population and differed in their policies on the sale of edible cannabis products. Alberta, British Columbia and Ontario allowed the sale of cannabis edibles after 2019 and Quebec did not. Before legalization, hospitalization rates were similar in the four provinces (0.95 per 100 000 person‐years in the first three versus 0.93 per 100 000 person‐years in Quebec). Hospitalization rates were 2.6 times higher in all provinces during the first period of legalization. In the second period, the hospitalization rate in Alberta, British Columbia and Ontario (7.15 per 100 00 person‐years) was 7.5 times higher than before legalization, and the hospitalization rate in Quebec (2.82 per 100 000 person‐years) was 3.0 times higher than before legalization. The increased hospitalizations for cannabis poisoning in children occurred after the legalization of cannabis edibles, despite regulations intended to reduce poisonings (e.g. setting a maximum of 10 mg of THC per edible package, plain and child‐resistant packaging and consumer education campaigns) [38].

#### Health service presentations

We summarized findings from five studies of presentations to hospital EDs for cannabis‐induced psychosis and mental health presentations, a study of cannabis‐positive cases postmortem in Coroner's cases and cannabis‐related hospitalizations [13, 39, 40, 41, 42]. Vignault *et al*. [42] assessed the short‐term impact of the legalization on cannabis use, cannabis use disorder and psychotic disorders among patients in one Quebec hospital before and 5 months after cannabis use was legalized. They analysed data on patients 12 years and older who visited a psychiatrist in the emergency unit of the hospital and compared trends in 1247 consultations in the 2 years before and 1368 consultations in the 5 months after legalization. Among those aged more than 18 years, there was a statistically significant increase in the reported use of cannabis (28–37%) and the prevalence of cannabis use disorder (18–24%). These increases were most marked in patients aged 18–24 years. There was no statistically significant increase in rates of psychotic diagnoses (27–29%).

Callaghan *et al*. [39] examined trends in ED presentations with an ICD diagnosis of cannabis‐induced psychosis or schizophrenia and related conditions in Alberta and Ontario between 1 April 2015 and 31 December 2019. ED presentations for cannabis‐induced psychosis doubled between April 2015 and December 2019, but there was no significant increase associated with cannabis legalization. The comparison of hospitalization rates before and after legalization did not take account of the fact that cannabis‐related psychiatric presentations were increasing before legalization of adult use. Maloney‐Hall *et al*. [41], for example, found that the rate of cannabis‐related hospitalizations for an ICD‐10 mental or behavioural disorder due to cannabis more than doubled between 2006 and 2015 (from 2.11 in 2006 to 5.18 per 100 000 in 2015). The increase was most marked in people aged 15–24 years (a 19‐fold increase between 2006 and 2015). This period included 2 years of data after the liberalization of medical cannabis laws in Ontario, which increased access to more potent cannabis products.

Kim *et al*. [40] examined trends in hospitalizations in Ontario for cannabis‐related diagnoses before legalization and during two phases after legalization. In Phase 1, only herbal cannabis flower was sold in a limited number of retail outlets. In Phase 2, cannabis edibles were sold from a much larger number of retail outlets. The study cohort (*n* = 12 079 699) included all adults aged 18+ in Ontario in the Ontario Health Insurance Plan in 17 October 2015. The most common cannabis diagnoses were harmful use (F12.1) and dependence (F12.2) accounted for 41 and 39% of hospitalizations, respectively. Adults aged 18–24 had higher rates of hospitalization than adults over 25 years in all three study phases.

Before legalization, there were significant monthly reductions in rates of cannabis‐related hospitalizations in women aged 18–24 and men aged 18–24. During Phase 1, there were significant immediate and monthly increases in hospitalization in all age groups and larger in men than women. There were no significant immediate or monthly increases in Phase 2 in any sex and age groups. The authors suggested that the increase in cannabis‐related hospitalizations after legalization may have reflected increased clinician attention to cannabis.

Jordan *et al*. [13] examined changes in the postmortem detection of cannabinoids in 3060 deaths in the province of New Brunswick before and after cannabis legalization between January 2014 and May 2020. They found that decedents after cannabis legalization (21%) were more likely to have cannabis present post‐mortem than those who died before legalization (17% present). The increase in cannabinoid‐positive cases was only significant in the 25–44‐year age group. There were greater proportions of cannabinoid‐positive samples after legalization in deaths that were judged to be suicidal (18.1–30.7%) or accidental (25.8–36.1%).

## DISCUSSION

In Table 1, we summarize the evidence on each of the study outcomes and the limitations of the studies and provide a summary statement on whether it is plausible that legalization was responsible for the changes in the outcome. We briefly state our conclusions in narrative form.

The legalization of cannabis use in Canada has greatly reduced cannabis arrests, as the Cannabis Act intended. It has also increased adult's legal access to a more diverse range of higher potency cannabis products, increased the proportion of cannabis purchases reportedly made from legal sources and greatly reduced cannabis prices. The first three were goals of legalization and all the changes observed are plausibly the result of policy.

Since legalization, the prevalence of recent cannabis use (e.g. in the previous 3 months) has increased among adults. There are limited survey data on whether the frequency of use has increased among existing users, and there is mixed survey evidence on whether cannabis use has increased among young people who are under the legal purchase age. Survey data may be affected by reduced reporting biases after legalization, but a modest increase in recent cannabis use among adults was an expected outcome of legalization.

There has not been a marked increase in traffic‐related injuries in Canada since legalization, and there is mixed evidence on changes in the prevalence of cannabis‐impaired driving. In surveys, fewer cannabis users report driving while impaired by cannabis, but analyses of blood THC levels in accident victims in British Columbia suggest that drivers in that Province are driving with higher blood THC levels since legalization.

The number of emergency presentations to hospitals for cannabis‐related reasons has increased among young adults since cannabis legalization. The increase was most marked after the commercialization of cannabis retail sales. These presentations have most often been for hyperemesis and symptoms of psychoses and cannabis use disorders. There have been increased numbers of children presenting to EDs after the accidental ingestion of cannabis products. This trend has been most discernible since the sale of edible cannabis products was allowed in 2019. The apparent increase in the number of cannabis‐related hospitalizations in adults in some provinces may be partly attributable to increased clinician questioning about cannabis use since legalization. Similar increases have also been observed in US states that have legalized cannabis [43].

### Limitations of the data

The outcomes of cannabis legalization in Canada that have been assessed have been limited to the first 6–24 months after legalization. The interpretation of trends in cannabis use and cannabis‐related harms after 2020 was complicated by the COVID pandemic and the social restrictions that were introduced to reduce its spread.

Canada has much less extensive and consistent survey data on cannabis use and cannabis use disorders than the United States. In the United States, surveys have shown an increase in the frequency of use among people who already used cannabis since legalization [44, 45], but conflicting results on the prevalence of cannabis use disorders [46]. US evaluations of the effects of legalization on cannabis impaired driving have also found conflicting results [45]. Studies in the United States and Canada have more consistently found increased ED attendance for acute adverse effects of cannabis in young adults and children [45].

The implementation of cannabis legalization in Canada is at an early stage. Cannabis production and legal sales are still increasing, so it would be unwise to assume that the modest short‐term adverse health effects of cannabis legalization will predict its public health effects [46] as a more commercialized cannabis industry develops over the next few decades. This is for several reasons: one should expect a delay between any increases in cannabis use and the detection of problems related to regular use; it will take longer before we can assess how much legalization has increased cannabis use and cannabis‐related harm in the Canadian population; and a profitable legal cannabis industry may succeed in lobbying for reductions in regulation and cannabis taxes intended to minimize heavy use and harms.

### Priorities for future evaluations of cannabis legalization in Canada

Future evaluations of the public health impacts of cannabis legalization in Canada need to more effectively monitor trends in the frequency and potency of cannabis use among youth and young adults. Studies should use shared standardized methods of assessing cannabis use and cannabis use‐related outcomes. Improved standardization of questions regarding cannabis use have been developed by Hammond *et al*. [47]. Surveys should also focus upon cannabis use in high‐risk populations, such as young people who seek help for mental health and drug use problems or who are involved in the criminal justice system.

Prospective studies of large representative samples of Canadians are needed that examine longer‐term changes in patterns of cannabis use and cannabis‐related harm (e.g. Bachman [48]). These studies should examine whether legalization extends the duration of cannabis use beyond the late 20s, the age at which most users ceased cannabis use under prohibition in the United States [48]. There is some suggestion that it may have done so in more recent US birth cohorts [49]. The health effects of daily cannabis use continued over decades of adult life may differ from those of more time limited daily use that begins in adolescence and ends in the mid to late 20s.

We need more accurate assessments of the contribution that cannabis‐impaired driving has made to fatal and non‐fatal car crashes since legalization. We also need analyses of longer‐term trends in ED attendances and hospitalizations for cannabis‐related acute and chronic adverse health effects, clearer data on treatment‐seeking for cannabis use disorders and clearer data on the prevalence of problem cannabis use among people with psychoses, major depression, anxiety and bipolar disorders [10].

The public health impact of cannabis legalization may depend upon how it affects the use and harms arising from other drugs that produce substantial public health harm; namely, alcohol, tobacco and opioids [50]. If, for example, cannabis proves to be a complement to alcohol, then the legalization of cannabis could increase the harm arising from alcohol. If, however, cannabis serves as a substitute for alcohol, then legalization could reduce alcohol‐related harm.

It will also be important to assess differential social impacts of legalization. Retail cannabis stores in Canada are concentrated in low socio‐economic status neighbourhoods [51]. If the greater number of cannabis retail outlets in these areas produces greater increases in cannabis use, then cannabis legalization may remove social inequities in rates of arrests while producing social inequities in the prevalence of cannabis use disorders in populations with poorer access to treatment. It would be important to quantify the impact of cannabis legalization on social inequities in each type of outcome.

A priority for cannabis policy research should be robust comparisons of the costs and benefits of cannabis policies within the different provincial and territories of Canada. These studies could, for example, assess the extent to which government retail monopolies and differences in the legal age of cannabis purchase affect youth uptake, rates of cannabis use and problems related to cannabis use in young adults [52].

As the legal cannabis industry grows, public health researchers will need to monitor industry activities to reduce public health‐orientated cannabis regulation. These studies could adopt some of the methods developed in studying the promotional and lobbying activities of the alcohol industry [53, 54].

## AUTHOR CONTRIBUTIONS

**Wayne D Hall:** Conceptualization (lead); formal analysis (lead); methodology (equal); project administration (lead); supervision (equal); writing—original draft (lead); writing—review and editing (lead). **Daniel Stjepanović:** Conceptualization (supporting); formal analysis (supporting); methodology (supporting); supervision (supporting); writing—original draft (supporting); writing—review and editing (supporting). **Janni Leung:** Conceptualization (supporting); data curation (lead); formal analysis (supporting); methodology (supporting); project administration (supporting); supervision (supporting); writing—original draft (supporting); writing—review and editing (supporting). **Danielle Dawson:** Data curation (supporting); methodology (supporting); validation (supporting); writing—review and editing (supporting).

## DECLARATION OF INTERESTS

The authors have none to declare.

## Supporting information


**Table S1:** Provincial cannabis policies.
**Table S2:** Canadian surveys of cannabis use.
**S3.** Search string used in systematic review of the public health impacts of cannabis legalization in Canada.
**Figure S4.** Flow diagram on identification of studies vias database and supplementary search.
**Figure S5.** Police‐reported drug offences per 100 000 population for cannabis, cocaine, and other drugs. Data provided by Moreau and colleagues [14].
**Figure S6.1.** Cumulative number of legal cannabis stores (top) and cannabis sales (bottom) by jurisdiction. Data provided by Roterman [16].
**Figure S6.2.** Cannabis sales by jurisdiction. Note: Q3 2021 and Q4 2021 sales data not reported for Northwest Territories, Prince Edward Island and Yukon. Figure reproduced from [10].
**Figure S6.3.** Proportion of total cannabis packaged units sold by product type. Data drawn from [18].
**Figure S6.4.** Household expenditure on cannabis sourced from the illegal recreational, legal recreational and medicinal markets. Data provided by Statistics Canada [14].
**Figure S7.** Prevalence of daily or near daily cannabis use over past three months. Data drawn from Rotermann and Macdonald [21].
**S7.** Quality assessment of studies based on the Joanna Briggs Institute (JBI) critical appraisal tool.
**S8.** Summary of individual studies on the impacts of cannabis legalization in Canada.

## Data Availability

The study used publicly available data and summarised the findings of published papers.
