# Supplementary material for: The implementation and public health impacts of cannabis legalization in Canada: a systematic review
Source: Addiction. 2023 Jun 28;118(11):2062–72. doi: 10.1111/add.16274 (PMC10953418; doi:10.1111/add.16274)
Supplement: Supplementary file 1 — Table S1: Provincial cannabis policies. Table S2: Canadian surveys of cannabis use. S3. Search string used in systematic review of the public health impacts of cannabis legalization in Canada. Figure S4. Flow diagram on identification of studies vias database and supplementary search. Figure S5. Police‐reported drug offences per 100 000 population for cannabis, cocaine, and other drugs. Data provided by Moreau and colleagues [14]. Figure S6.1. Cumulative number of legal cannabis stores (top) and cannabis sales (bottom) by jurisdiction. Data provided by Roterman [16]. Figure S6.2. Cannabis sales by jurisdiction. Note: Q3 2021 and Q4 2021 sales data not reported for Northwest Territories, Prince Edward Island and Yukon. Figure reproduced from [10]. Figure S6.3. Proportion of total cannabis packaged units sold by product type. Data drawn from [18]. Figure S6.4. Household expenditure on cannabis sourced from the illegal recreational, legal recreational and medicinal markets. Data provided by Statistics Canada [14]. Figure S7. Prevalence of daily or near daily cannabis use over past three months. Data drawn from Rotermann and Macdonald [21]. S7. Quality assessment of studies based on the Joanna Briggs Institute (JBI) critical appraisal tool. S8. Summary of individual studies on the impacts of cannabis legalization in Canada. [file ADD-118-2062-s001.docx]

# Supplementary appendix

# The implementation and public health impacts of cannabis legalisation in Canada – a systematic review (2023)

Wayne Hall, Daniel Stjepanović, Danielle Dawson, and Janni Leung

National Centre for Youth Substance Use Research

The University of Queensland, Australia

Table of contents

[The implementation and public health impacts of cannabis legalisation in Canada – a systematic review (2023) 1](#_Toc128412285)

[S1. Provincial cannabis policies of Canada in 2022 (post legalization) 2](#_Toc128412286)

[S2. Main Canadian population surveys that have reported data on cannabis use in the context of legalisation 3](#_Toc128412287)

[S3. Search string used in systematic review of the public health impacts of cannabis legalisation in Canada 4](#_Toc128412288)

[S4. PRISMA 2020 flow diagram for the systematic search 5](#_Toc128412289)

[S5. Cannabis-related arrests 6](#_Toc128412290)

[S6. Cannabis prices and markets 7](#_Toc128412291)

[S7. Quality assessment of studies based on the Joanna Briggs Institute (JBI) critical appraisal tool 0](#_Toc128412292)

[S8. Summary of individual studies on the impacts of cannabis legalisation in Canada 0](#_Toc128412293)

**Note.** The periods of pre- and post-legalization were:

Pre-legalization: before 17 Oct 2018

Phase 1 / peri-legalization: 17 Oct 2018, legal sales were limited to dried cannabis, fresh cannabis, cannabis oil, cannabis plants, and cannabis seeds.

Phase 2 / post-legalization: 17 Oct 2019 onwards, the sale of edible cannabis, cannabis extracts, and cannabis topicals was permitted, regulations were amended to include controls to protect public health and cannabis-related risks.

# S1. Provincial cannabis policies of Canada in 2022 (post legalization)

## Table S1: Provincial cannabis policies

| **Province** | **Retail models** | **N Stores per 10,000** | **Grow your own** | **Use in public** | **Sale with alcohol** | **Age of purchase** | **Edibles and extracts** |
| --- | --- | --- | --- | --- | --- | --- | --- |
| Alberta | Private in-person & govt online | 599 | yes | no | no | 18 | yes |
| British Columbia | Private in-person and govt in-person and online | 356 | yes | no | no | 19 | yes |
| Manitoba | Private in-person and online | 107 | no | no | no | 19 | yes |
| New Brunswick | Govt in-person and online | 20 | yes | no | no | 19 | yes |
| Newfoundland and Labrador | Private in-person and govt online | 30 | yes | no | no | 19 | yes |
| Nova Scotia | Govt in-person and online | 30 | yes | no | no | 19 | yes |
| Ontario | Private in person & govt online | 613 | yes | no | no | 19 | yes |
| Prince Edward Island | Govt in-person and online | 4 | yes | no | no | 19 | yes |
| Québec | Government stores only | 65 | no | no | no | 21 | no |
| Saskatchewan | Private in person and online | 80 | yes | no | no | 19 | yes |

# S2. Main Canadian population surveys that have reported data on cannabis use in the context of legalisation

## Table S2: Canadian surveys of cannabis use

| **Name of survey** | **When conducted** | **Age**  **range** | **Key measures of cannabis use** | **N** | **Method** |
| --- | --- | --- | --- | --- | --- |
| National Cannabis Survey | Quarterly since 2018 to 2020 | 15+ | Use in past 3 months  Daily use  Source of cannabis  Driving after using cannabis | 5400-6200 | Online |
| Canadian Cannabis Survey | Annual from 2017 to 2020. | 16 + | Use in past 12 or 1 month  Daily use  Driving after using cannabis  Source of cannabis  Perception of health risks | 11,000 | Online |
| Canadian Student Tobacco, Alcohol and Drugs Survey | 2014-15  2016-17  2018-19 | 12-18 | Use in past 12 months  Method of use  Perceived health risks | 62,000 | Pencil and paper and  interviews |
| International Cannabis Policy Study | Annually since 2018 | 16-65 | Use within different  time periods  Methods of use  Quantity used  Daily or near daily use  Cannabis-impaired driving  Sources of cannabis | 10,000-15,000 | Online |

Note. Pre-legalization: before 17 Oct 2018, Phase 1 / peri-legalization: 17 Oct 2018 – 16 Oct 2019, Phase 2 / post-legalization: 17 Oct 2019 onwards

# S3. Search string used in systematic review of the public health impacts of cannabis legalisation in Canada

(accidents, traffic [MeSH Terms] OR cannabis-impaired drivers [Title/Abstract] OR cannabis-impaired driving [Title/Abstract] OR cannabis-intoxicated drivers [Title/Abstract] OR cannabis-intoxicated driving [Title/Abstract] OR Motor vehicle accident* [Title/Abstract] OR MVA [Title/Abstract] OR MVAs [Title/Abstract] OR car crash*[tiab] OR car accident*[tiab] OR motor vehicle crash*[tiab] OR traffic crash*[tiab] OR traffic accident*[tiab] OR fatal* [Title/Abstract] OR injur* [Title/Abstract] OR Death, Sudden, Cardiac [MeSH Terms] OR Drug-Related Side Effects and Adverse Reactions [MeSH Terms] OR Emergency Service, Hospital [MeSH Terms] OR Hospitalization [MeSH Terms] OR Hyperemesis Gravidarum [MeSH Terms] OR Neoplasms [MeSH Terms] OR Poisoning [MeSH Terms] OR Residential Treatment [MeSH Terms] OR Substance Abuse Treatment Centers [MeSH Terms] OR Pregnancy Outcome [MeSH Terms] OR Vomiting [MeSH Terms]) AND (Canada [MeSH Terms] OR Canada [Title/Abstract]) AND (legalisation [Title/Abstract] OR legalization [Title/Abstract] OR legislation [Title/Abstract] OR decriminalization [Title/Abstract] OR decriminalisation [Title/Abstract] OR Medical marijuana laws [Title/Abstract] OR Medical marijuana law [Title/Abstract] OR MML [Title/Abstract] OR MMLs[Title/Abstract] OR medical cannabis laws [Title/Abstract] OR medical cannabis laws [Title/Abstract] OR policy [Title/Abstract] OR policies [Title/Abstract] OR regulation [Title/Abstract] OR regulations [Title/Abstract] OR Legislation as Topic [MeSH Terms] OR Legislation, Medical [MeSH Terms] OR Jurisprudence [MeSH Terms] OR Legislation, Drug [MeSH Terms] OR Government Regulation [MeSH Terms]) AND (cannabis[Title/Abstract] OR marijuana[Title/Abstract] OR Cannabis[MeSH Terms] OR Marijuana Use[MeSH Terms] OR Medical Marijuana[MeSH Terms])

# S4. PRISMA 2020 flow diagram for the systematic search


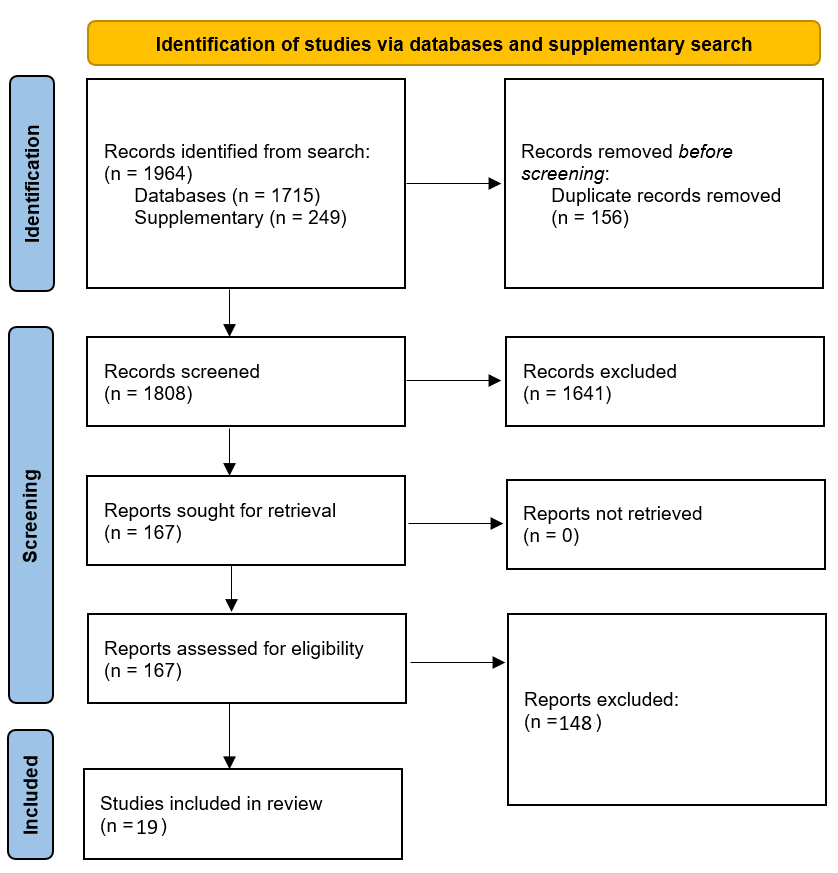


## Figure S4. Flow diagram on identification of studies vias database and supplementary search

# S5. Cannabis-related arrests


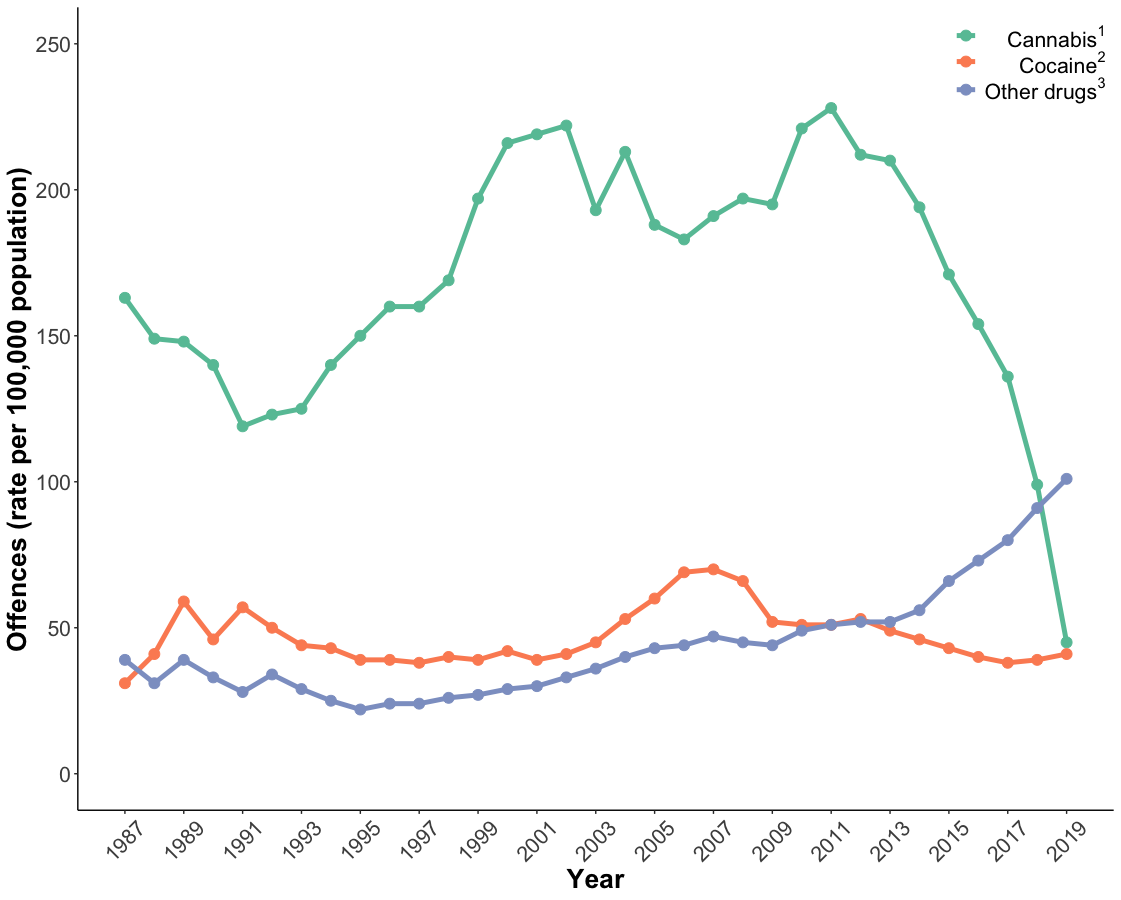


## Figure S5. Police-reported drug offences per 100,000 population for cannabis, cocaine, and other drugs. Data provided by Moreau and colleagues (14).

**N.B.** ^1^Cannabis offences include those that fall under the *Controlled Drugs and Substances Act* prior to 17 October 2018. Following 17 October 2018 cannabis offences include those falling under the *Cannabis Act*. Offences include possession, trafficking, importation or exportation, production, distribution, sale and “other” cannabis violations.
^2^Offences include possession, trafficking, production, importation and exportation.
^3^Other drugs includes heroin, methamphetamines (including crystal), methylenedioxyamphetamine (MDMA or ecstasy), opioids (including fentanyl but excluding heroin) and other controlled drugs and substances, as well as possession of precursors and equipment.

# S6. Cannabis prices and markets


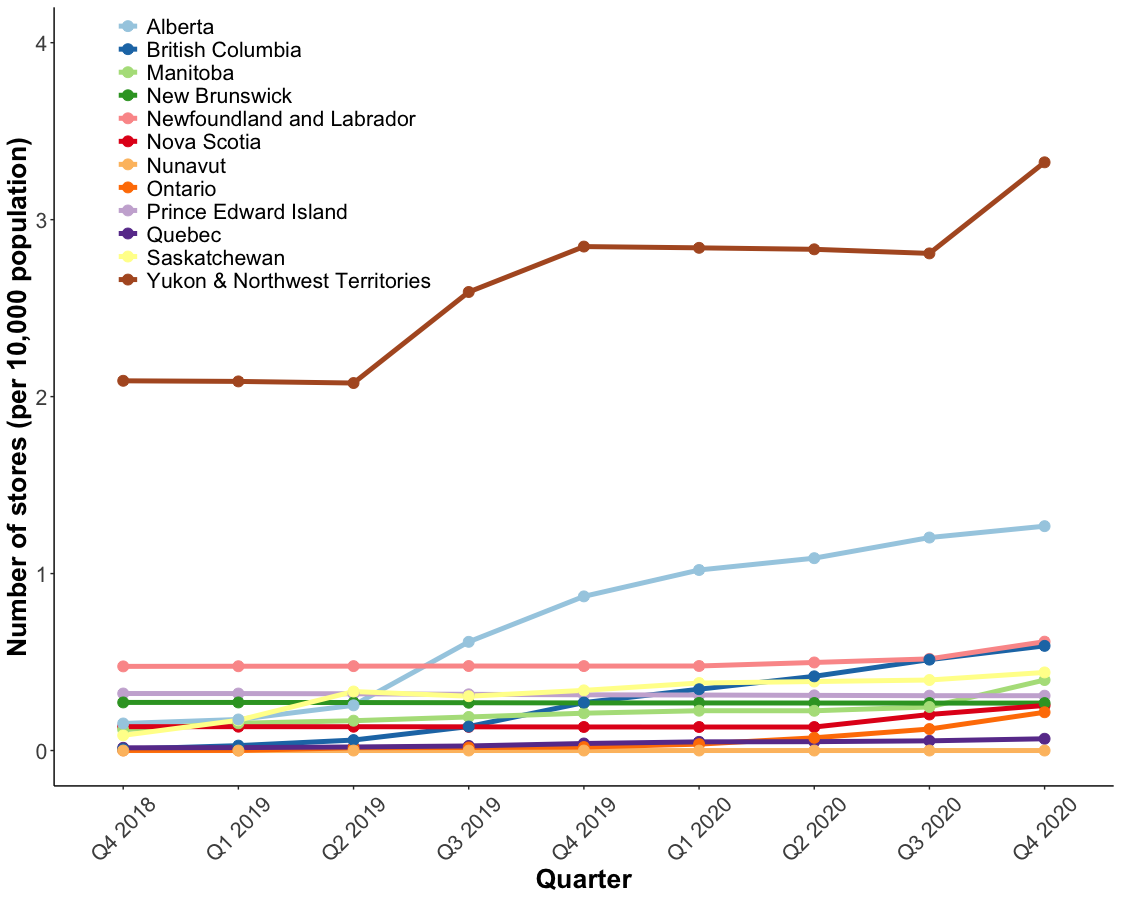


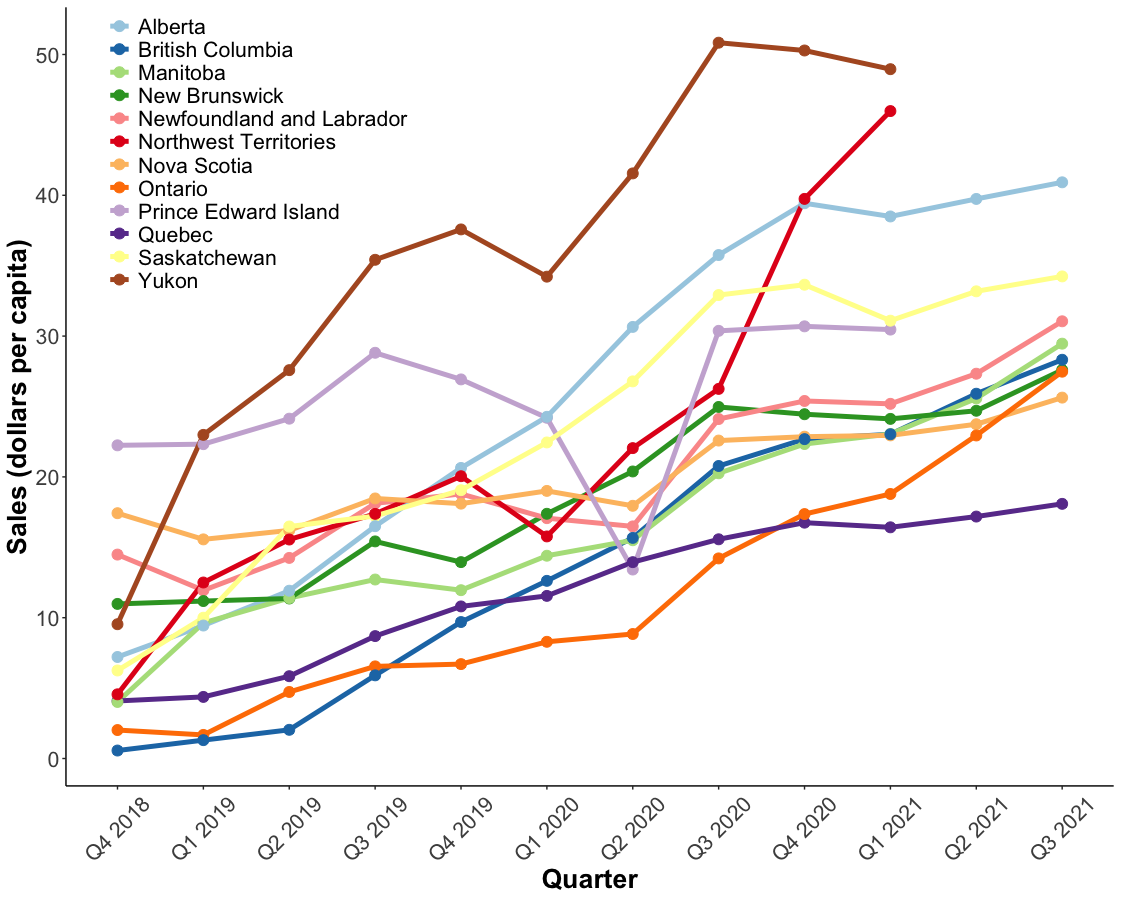


## Figure S6.1. Cumulative number of legal cannabis stores (top) and cannabis sales (bottom) by jurisdiction. Data provided by Roterman (16).

Note: Yukon and Northwest Territories are combined when reporting the number of legal cannabis stores (top). Pre-legalization: before 17 Oct 2018, Phase 1 / peri-legalization: 17 Oct 2018 – 16 Oct 2019, Phase 2 / post-legalization: 17 Oct 2019 onwards. Dates for Q1: January 1 – March 31, Q2: April 1 – June 3, Q3: July 1 – September 30, Q4: October 1 – December 31.

## Figure S6.2. Cannabis sales by jurisdiction. Note: Q3 2021 and Q4 2021 sales data not reported for Northwest Territories, Prince Edward Island and Yukon. Figure reproduced from (10).

*Note. Pre-legalization: before 17 Oct 2018, Phase 1 / peri-legalization: 17 Oct 2018 – 16 Oct 2019, Phase 2 / post-legalization: 17 Oct 2019 onwards.*


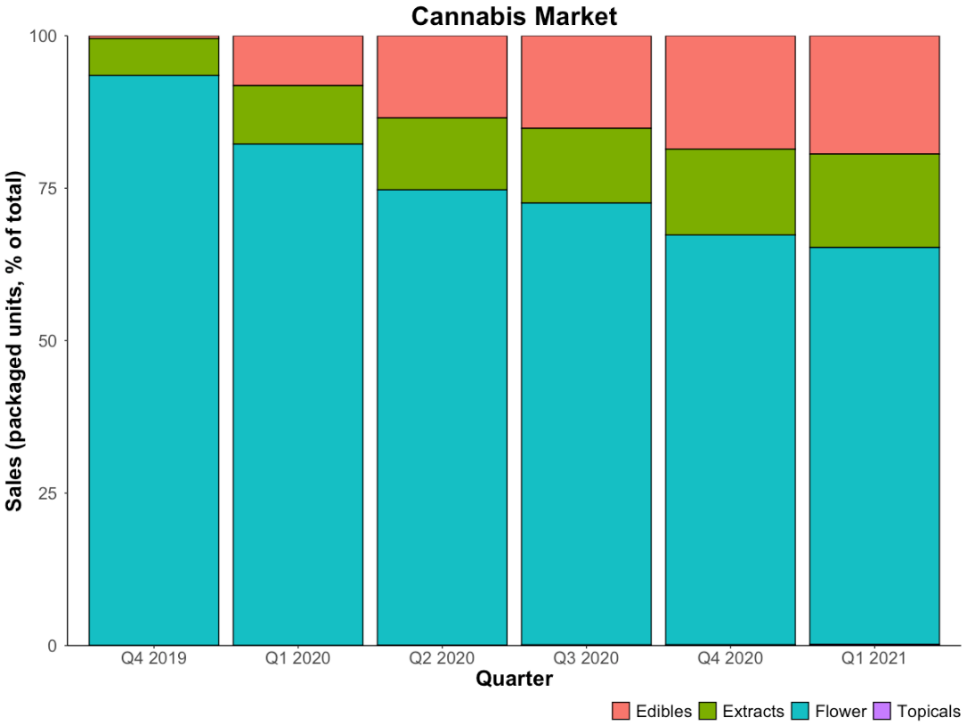


## Figure S6.3. Proportion of total cannabis packaged units sold by product type. Data drawn from (18).

*Note.* Pre-legalization: before 17 Oct 2018, Phase 1 / peri-legalization: 17 Oct 2018 – 16 Oct 2019, Phase 2 / post-legalization: 17 Oct 2019 onwards. Dates for Q1: January 1 – March 31, Q2: April 1 – June 3, Q3: July 1 – September 30, Q4: October 1 – December 31.


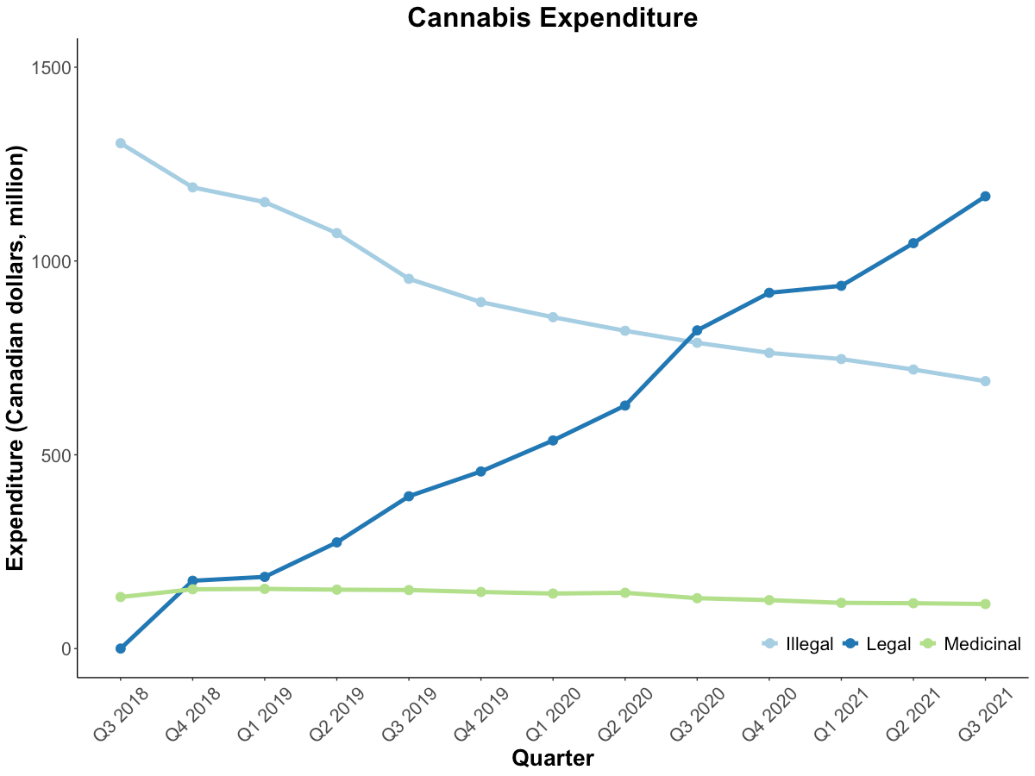


## Figure S6.4. Household expenditure on cannabis sourced from the illegal recreational, legal recreational and medicinal markets. Data provided by Statistics Canada (14).

*Note.* Pre-legalization: before 17 Oct 2018, Phase 1 / peri-legalization: 17 Oct 2018 – 16 Oct 2019, Phase 2 / post-legalization: 17 Oct 2019 onwards. Dates for Q1: January 1 – March 31, Q2: April 1 – June 3, Q3: July 1 – September 30, Q4: October 1 – December 31.


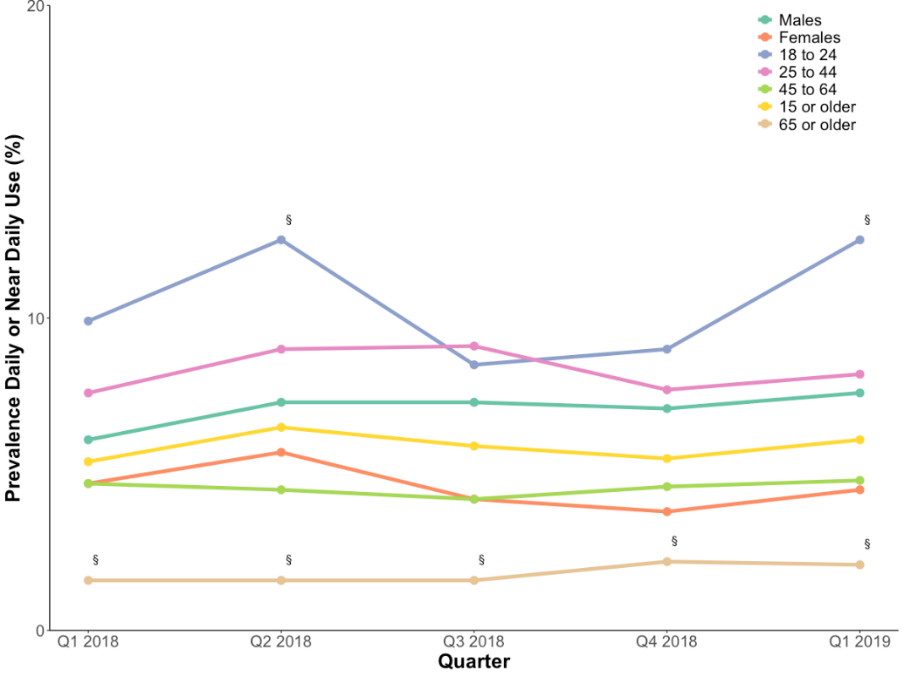


## Figure S7. Prevalence of daily or near daily cannabis use over past three months. Data drawn from Rotermann and Macdonald (21).

**N.B.** Linear trends are not statistically significant (*p* > .05). § This data point should be interpreted with caution.

*Note.* Pre-legalization: before 17 Oct 2018, Phase 1 / peri-legalization: 17 Oct 2018 – 16 Oct 2019, Phase 2 / post-legalization: 17 Oct 2019 onwards. Dates for Q1: January 1 – March 31, Q2: April 1 – June 3, Q3: July 1 – September 30, Q4: October 1 – December 31.

# S7. Quality assessment of studies based on the Joanna Briggs Institute (JBI) critical appraisal tool

| Study | Criteria for inclusion in the sample clearly defined | Study subjects and the setting described in detail | Exposure measured in a valid and reliable way | Objective, standard criteria used for measurement of the condition | Confounding factors identified | Strategies to deal with confounding factors stated | Outcomes measured in a valid and reliable way | Appropriate statistical analysis used | Total  (out of 8) |
| --- | --- | --- | --- | --- | --- | --- | --- | --- | --- |
| Auger 2021 | Yes | Yes | Yes | Yes | Yes | No | Yes | Yes | 7 |
| Baraniecki 2021 | Yes | Yes | Yes | Yes | Yes | Yes | Yes | Yes | 8 |
| Brubacher 2022 | Yes | Yes | Yes | Yes | Yes | Yes | Yes | Yes | 8 |
| Callaghan 2021 | Yes | Yes | Yes | Yes | Yes | No | Yes | Yes | 7 |
| Cohen (2022) | Yes | Yes | Yes | Yes | Yes | No | Yes | Yes | 7 |
| Coret (2022) | Yes | Yes | Yes | Yes | Yes | Yes | Yes | No | 7 |
| Fischer (2021) | Yes | Yes | Yes | No | Yes | No | Yes | Yes | 6 |
| Jordan 2022 | Yes | Yes | Yes | Yes | Yes | No | Yes | Yes | 7 |
| Kim (2022) | Yes | Yes | Yes | Yes | No | No | Yes | Yes | 6 |
| Maloney-Hall (2020) | Yes | Yes | Yes | Yes | Yes | No | Yes | Yes | 7 |
| Morean (2022) | Yes | Yes | Yes | Yes | Yes | No | Yes | No | 6 |
| Myran (2022a) | Yes | Yes | Yes | Yes | Yes | No | Yes | Yes | 7 |
| Myran (2022b) | Yes | Yes | Yes | Yes | Yes | No | Yes | Yes | 7 |
| Myran 2023 | Yes | Yes | Yes | Yes | Yes | No | Yes | Yes | 7 |
| Pham (2022) | Yes | Yes | Yes | Yes | Yes | No | No | Yes | 6 |
| Vignault (2021) | Yes | Yes | Yes | Yes | Yes | No | Yes | Yes | 7 |
| Yeung 2020 | Yes | Yes | Yes | Yes | Yes | No | Yes | Yes | 7 |
| Yeung 2021 | Yes | Yes | Yes | Yes | Yes | No | Yes | Yes | 7 |

# S8. Summary of individual studies on the impacts of cannabis legalisation in Canada

| **Study first author (year): aim [Quality]** | **Design & Setting; year of data** | **Outcome** | **Measure** | **Estimates** | **Impact** | **Key results and conclusions** |  |
| --- | --- | --- | --- | --- | --- | --- | --- |
| **Cannabis-related arrests** | | | | | | | |
| Moreau (2022): To compare rates of police-reported crime statistics over time [Q=6] | Police-reported crime statistics, 1987-2019 | Drug offence rate per 100k population | Cannabis-related arrests per 100k | >200 in 2010;  <100 in 2017;  <50 in 2019 | - | There was a sharp decline in the number of persons arrested for cannabis possession and use after the legalisation of adult use. The decline began before legalisation, which may be because police gave a lower priority to making arrests after legislation to legalise adult use passed in 2017. |  |
| **Cannabis prices and markets** | | | | | | | |
| Fischer (2021): To summarise cannabis use and supply indicators pre- and post-legalisation in Canada [Q=6] | The National Cannabis Survey (NCS), household survey, aged 15+, N~12,000; 2018, 2019, 2020 | Cannabis sourcing from 2018 to 2019 to 2020 | Obtained from illegal sources | 2018=51.3%  2019=38.1%  2020=35.4% | - | In the NCS, obtaining cannabis from illegal sources or family and friends decreased, while obtaining from legal sources / growing cannabis increased. |  |
|  |  |  | Obtained from friends and family | 2018=47%  2019=37%  2020=28.6% | - |  |  |
|  |  |  | Obtained from legal sources | 2018=22.9%  2019=47.4%  2020=68.4% | + |  |  |
|  |  |  | Grew own or had it grown for them | 2018=8%  2019=9%  2020=14.2% | + |  |  |
|  | The Canadian Cannabis Survey (CCS), online survey, age 16+, N>10,000; 2018, 2019, 2020 |  | Legal storefront | 2018=24.5%  2020=40.6% | + | In the CCS, sourcing cannabis from legal storefronts increased; sourcing from legal online source did not. |  |
|  |  |  | Legal online store | 2018=12.8%  2020=13.3% | ns |  |  |
|  | The International Cannabis Policy Study (ICPS), web-based survey, age 16-65; 2018-2019 |  | Legal store | 2019=47.70% | n/a | In fall 2019, 47.7% of Canadian adult purchasers of dried cannabis flower products purchased from a legal source. |  |
|  | The Canadian Student Tobacco, Alcohol, and Drugs Survey (CSTADS), students in grades 7-12 (ages 13-18), N~45,000; 2016/17, 2018/19 |  | Easy to obtain | 2016/17=39%  2018/19=40% | ns | In CSTADS’ school students, reports that it would be easy to obtain cannabis remained stable; reports that it would be difficult to obtain cannabis decreased. |  |
|  |  |  | Difficult to obtain | 2016/17=46%  2018/19=42% | - |  |  |
|  | Ontario Cannabis Store (OCS); Q4/2018, Q4/2019, Q3/2020 |  | Sales from legal sources | 2018=4.6%  2019=24.7%  2020=40.3% | + | Cannabis market data from the OCS found that the share of cannabis sales from legal sources in Ontario increased. |  |
| Health Canada: To provide data for cannabis in the legal market [Q=7] | Cannabis market data; 2018-2021 | Market data | Number of legal cannabis stores | Alberta >1 per 10k population;  Other provinces 0-0.6;  Yukon & Northwest Territories >3 | + | Alberta had the fastest rate of increase in the number of outlets that opened, other provinces initially licensed a small number and have not licenced many more since then. The volume of cannabis sales has steadily increased in all jurisdictions since legalisation. |  |
|  |  |  | Cannabis Sales | 2018=$0-$20  2021=$15-$50 per capita | + |  |  |
|  |  |  | Cannabis prices | Quebec  2018=$7.9  2019=$8.9 2021=$8.2  Other provinces 2018=$11.2  2019=$8.2  2021=$9.5 | - | Cannabis prices declined in the first two years after cannabis legalisation in most jurisdictions. Québec began selling cannabis in government stories at a much lower price then increased before declining in the second year of legalisation, which remained well below prices in other jurisdictions. |  |
|  |  | Proportion of sales (packaged units, % of total) | Edibles | 2018=1%  2021=20% | + | Edibles and extracts have accounted for an increasing proportion of total cannabis sales nationally in the two years after their sales commenced. |  |
|  |  |  | Extracts | 2018=<10%  2021=15% | + |  |  |
|  |  |  | Flower | 2018=>85%  2021=65% | - |  |  |
|  |  | Cannabis expenditure (Canadian dollars, million) | Illegal market | 2018=$1300  2021=$700 | - | Cannabis expenditure increased in the legal market, decreased in the illegal market, and remained very low in the medicinal market. |  |
|  |  |  | Legal market | 2018=$0  2021=$1250 | + |  |  |
|  |  |  | Medicinal market | 2018-21= ~$150 | ns |  |  |
| **Patterns of cannabis use** | | | | | | | |
| Fischer (2021): To summarise cannabis use and supply indicators pre- and post-legalisation in Canada [Q=6] | The National Cannabis Survey (NCS), household survey, aged 15+, N~12,000; 2018, 2019, 2020 | Prevalence of cannabis use | Cannabis use in past 3 months | 2018=14.9%  2019=16.8%  2020=20% | + | In the NCS, prevalence of cannabis use increased between 2018-2020; highest prevalence in young adults aged 18-24; no significant increase was observed in adolescents aged 15-17. |  |
|  | The Canadian Cannabis Survey (CCS), online survey, age 16+, N>10,000; 2018, 2019, 2020 |  | Cannabis use in past year | 2018=21.9%  2019=24.6%  2020=26.9% | + | In the CCS, prevalence of cannabis use increased; highest prevalence in young adults aged 20-24; prevalence increased in those aged 16-19. |  |
|  |  |  |  | 2018=5.5%  2020=7% in aged 13-15 | + |  |  |
|  | The Canadian Student Tobacco, Alcohol, and Drugs Survey (CSTADS), students in grades 7-12 (ages 13-18), N~45,000; 2016/17, 2018/19 |  | Cannabis use in past year | 2016/17=27.8%  2018/19=29.4%  in grades 10-12 | ns | In the CSTADS, past year cannabis use increased in grades 7-9, but did not increase significantly in grades 10-12. |  |
|  | The Centre for Addiction and Mental Health (CAMH) Monitor, telephone-interview cross-sectional household survey, age 18+ in Ontario; 2017, 2019 |  | Cannabis use in past year | 2017=19.4%  2019=25.6% | + | In the CAMH Monitor, prevalence of past year cannabis use increased; highest prevalence was in young adults aged 18-29. |  |
|  | The National Cannabis Survey (NCS), household survey, aged 15+; N~12,000; 2018, 2019, 2020 | Frequent cannabis use among users | Daily/near-daily use among users | 2018=39.6%  2019=35.7%  2020=39.5% | ns | In the NCS, ‘daily/near-daily’ use of cannabis among existing users did not increase. |  |
|  | The Canadian Cannabis Survey (CCS), online survey, age 16+, N>10,000; 2018, 2019, 2020 |  | Daily/near-daily use among users | 2018=24.9%  2019=23.9%  2020=24.8% | ns | In the CCS, proportion of daily/near-daily use among users remained unchanged. |  |
|  | The Centre for Addiction and Mental Health (CAMH) Monitor; 2017, 2019 |  | Moderate/high-risk ASSIST-CIS score | 2017=53.3%  2019=57.9% | + | In the CAMH Monitor, proportion of users with moderate or high-risk of cannabis use problems score on the ASSIST-CIS increased from 2017-2019 but with overlapping confidence intervals. |  |
| Pham (2022): To understand how cannabis use has changed post-legalization [Q=6] | Canadian Tobacco, Alcohol, and Drugs Survey (CTADS); n = 16,349 pre, n = 8,614 post; 2017 and 2019 | Prevalence of cannabis use | Cannabis use in past 30-days | 2017=9%  2019=11% | ns | The cannabis items in the 2017 and 2019 surveys differed in their wording, an approximate measure of use in the past 30-days was compared. On this measure, cannabis use increased from 9% in 2017 to nearly 11% (95% CI: 10.1, 11.7) in 2019 but it was not possible to confirm that this increase was statistically significant. There were few differences in prevalence between provinces in 2019, despite their different regulatory approaches. |  |
| **Driving after cannabis use** | | | | | | | |
| Brubacher (2022): To assess the effect of cannabis legalization on the prevalence of injured drivers testing positive for THC in British Columbia [Q=8] | Data from treated injured drivers from four British Columbia trauma centers, N=4339 (N=3550 before legalization, 789 after), Jan 2013-Mar 2020 | Testing positive for THC in moderately injured drivers | THC >0 | 9.2% pre vs 17.9% post;  PR=1.33 [1.05-1.68] | + | After legalization, there was an increased prevalence of THC detected in blood of treated injured drivers. The increase was largest among older drivers and male drivers. |  |
|  |  |  | THC >=2ng | 3.8 % pre vs 8.6% post;  PR=2.29 [1.52-3.45] | + |  |  |
|  |  |  | THC >=5ng | 1.1% pre vs 3.5% post; PR=2.05 [1.00-4.18] | + |  |  |
| Callaghan (2021): To assess cannabis legalization and drivers' traffic-injury presentations to emergency departments In Canada [Q=7] | Provincial emergency department (ED) records (Apr 2015-Dec 2019), Alberta (all n=52,752; youth n=3265) and Ontario (all n=186,921; youth n=4565) drivers | Weekly provincial counts of ICD-10-CA-defined traffic-injury ED presentations | Alberta, all | +9.17 [-18.85, 37.20], p=0.52 | ns | There were no significant post-legalization changes in traffic-injury ED visits in Ontario or Alberta among all drivers or youth drivers. |  |
|  |  |  | Alberta, youth | -0.66 [-2.2, 0.94], p=0.42 | ns |  |  |
|  |  |  | Ontario, all | +28.93 [-26.32, 84.19], p=0.30 | ns |  |  |
|  |  |  | Ontario, youth | +0.09 [-6.25, 6.42], p=0.98 | ns |  |  |
| Fischer (2021): To summarise cannabis use and supply indicators pre- and post-legalisation in Canada [Q=6] | The National Cannabis Survey (NCS), household survey, aged 15+, N~12,000; 2018, 2019, 2020 | Driving after cannabis use | Driving after cannabis use | 2018=14.2%  2020=13.2% | ns | In the NCS, the prevalence of individuals who reported driving a vehicle within 2 hours of cannabis consumption in past 3 months did not change. |  |
|  | The Canadian Cannabis Survey (CCS), online survey, age 16+, N>10,000; 2018, 2019, 2020 |  |  | 2018=27%  2019=24%  2020=19% | - | In the CCS, the proportion of respondents reporting that they drove after cannabis use in the past year decreased. |  |
|  | The Centre for Addiction and Mental Health (CAMH) Monitor; 2017, 2019 |  |  | 2017=13.4%  2019=12.1% | - | In the CAMH Monitor, cannabis users who self-reported driving within one hour of use decreased. |  |
| **Emergency department visits** | | | | | | | |
| Auger (2021): To compare cannabis-related hospitalizations in youth before and after cannabis legalization [Q=7] | Hospital records of youth aged 10-19 years in Quebec; 2017-19 | Cannabis-related presentations | Hospitalization rates per 100k: boys 10-14 | 2017=5.2  [2.9-9.3]  2018=9.5  [6.2-14.6] | ns | In boys aged 10 to 14 years, cannabis-related hospitalization increased, but were not statistically significant. The wide confidence intervals around the estimates suggests that an increase may not have been detected because of the small numbers of hospitalisations. No increases were observed in girls and in boys aged 15-19. |  |
|  |  |  | Cannabis reporting in substance-related hospitalizations: boys 10-14 | 2017=39.3%  [23.6-57.6]  2018=70.0%  [52.1-83.3] | ns |  |  |
|  |  |  | Cannabis presentations in girls or boys aged 15-19 | No increase | ns |  |  |
| Baraniecki (2021): To investigate how legalisation of cannabis has impacted emergency department visits for acute cannabis intoxication [Q=8] | Chart review of an emergency departments in Ontario, Hamilton, N=64,152 visits, N=173 attributed to acute cannabis intoxication; 17-Apr-2018 to 17-Apr-2019 | Cannabis-related ICD-10 discharge code | Acute cannabis intoxication ED visit rate - overall | 2.44 before vs 2.94 after/1000, p=0.27 | ns | Overall rate of cannabis-related ED visits did not increase after legalisation overall, cases of visits increased in young adults aged 18-29 but not in other age groups. |  |
|  |  |  | Cases of cannabis intoxication ED visit rate of young adults (aged 18-29) | 41 cases (52%) pre, 64 cases (68%) post, +56%, p=0.03 | + |  |  |
| Cohen (2022): To compare pediatric cannabis intoxication trends pre and post-legalization [Q=7] | A retrospective cohort study of children 0-18 years of a pediatric ED presenting with cannabis intoxication (N=298, pre n=232, post n=66), 2008 to 2019; pre-legalization 1 Jan 2008 to 12 Apr 2017, peri-post 13 Apr 2017 to 31 Dec 2019 | ICU admission rates of children presenting with cannabis intoxication | Median monthly number of cannabis-related presentations | 2.1 [IQR:1.9-2.5] pre to 1.7 [IQR:1.0-3.0] post, p=.69 | ns | Post-legalization of cannabis for recreational use was associated with increased rates of cannabis-related presentations indicative of more severe intoxications in younger children. Edible ingestion was a strong predictor of ICU admission. |  |
|  |  |  | Proportion of children admitted to the ICU | 13.6% vs. 4.7% pre to 13.6% post, p=.02 | + |  |  |
|  |  |  | Proportions of children <12 years | 3.0% pre to 12.1% post, p=.04 | + |  |  |
|  |  |  | Proportions with respiratory involvement | 50.9% pre to 65.9% post, p=.05 | + |  |  |
|  |  |  | Proportions with altered mental status | 14.2% pre to 28.8% post, p<.01 | + |  |  |
|  |  |  | Proportions involving unintentional exposures | 2.8% pre to 14.4% post, p=.002 | + |  |  |
|  |  |  | Proportions involving edibles ingestion | 7.8% pre to 19.7% post, p=.01 | + |  |  |
| Coret (2022): To compare unintentional cannabis exposure in children pre- and post-legalization [Q=7] | A retrospective chart review of Emergency Department visits at the Children's Hospital of Eastern Ontario (Ottawa, ON), N=37 children, mean age 5.9±3.8 years; March 2013 to Sept 2020 | ED visits of unintentional cannabis ingestion (ICD-10 codes T40.7 and X42) | Unintentional ingestion, number of visits | 5 visits (14%) pre to 32 visits (86%) post | + | Most visits occurred in the 2-year period after legalization. The most common symptoms were altered levels of consciousness, lethargy or somnolence, tachycardia, and vomiting. 76% of cases were exposed to edibles . A third (32%) required admission to the hospital for less than 24 hours. |  |
|  |  |  | Proportion of exposures to edibles | 76% |  |  |  |
|  |  |  | Proportion of exposures in home setting | 81% |  |  |  |
|  |  |  | Proportion requiring hospital admission | 32% |  |  |  |
| Myran (2022): To examine changes in emergency department visits and hospitalizations due to cannabis exposures among children after legalization of recreational cannabis in Canada [Q=7] | ED visits in Ontario, children aged 0-9 years, N=522 ED visits due to cannabis exposures, N=81 before legalisation, N=124 visits in the first period, and N=317 visits in the second phase of legalisation; 2016-2021 | Proportion of ED visits requiring hospitalization | Cannabis-related visits | IRR=3.13 in 1st period, IRR=9.12 in 2nd period | + | The proportion of cannabis-related ED visits requiring hospitalization increased significantly after edibles were sold (122 vs 29 in the first period compared with 20 before legalization). Rates of cannabis-related ED visits increased from January 2016 to March 2021 in both the first (IRR, 3.13) and second period (IRR, 9.12). After adjusting for trends in cannabis-related ED visits throughout the whole study period, there was an increase in visits during the period when edibles could be sold (IRR, 2.23). |  |
| Myran (2022): To compare commercialization and cannabis-attributable emergency department visits after cannabis legalization [Q=7] | Interrupted time-series analysis of emergency department visits in Ontario, N=13.8 million individuals, age 15-105; 2016-2021 | Trends of cannabis-attributable ED visits across periods 1) RCL: with store restrictions and 2) RCC: no store restrictions | Cannabis-attributable visits in RCL | +12%, IRR=1.12 [1.02-1.23],  then IRR=0.98 [0.97-0.99] | + then - | Cannabis attributable ED visits were increasing before legalisation but there was a small statistically significant 18% decline in the monthly visit rate when sales were limited to online, then significantly increased immediately after commercialised retail sales. |  |
|  |  |  | Cannabis-attributable visits in RCC | +22%, IRR=1.22 [1.09-1.37] | + |  |  |
|  |  |  | Proportion of all-cause ED visits attributable to cannabis | +17%, IRR=1.17 [1.00-1.37] | + |  |  |
| Myran (2023): To compare sale of edible cannabis products and cannabis-attributable hospitalizations for paediatric cannabis poisonings [Q=7] | Paediatric hospitalisation in Ontario, Quebec, Alberta, and British Columbia  children aged 0-9 years, N= 581 due to cannabis poisoning, N=120 before legalisation (2015-2018), N=105 visits in the first period (2018-2019), and N=356 visits in the second phase of legalisation; 2020-2021 | Proportion of cannabis poisonings requiring hospitalization | Cannabis-related hospitalisation | IRR= 2.55; [1.88-3.46] in 1st period, IRR= 2.16; [1.68-2.80]  in 2nd period | + | The proportion of paediatric cannabis poisonings requiring hospitalization increased significantly in provinces that permitted edible cannabis sales compared to control provinces over three periods (pre-legalisation (January 2015 – September 2018), period 1 [October 2018 – December 2019], period 2 [January 2020 – September 2021]). |  |
| Yeung (2020): To examine changes in cannabis-related ED visits pre- and post-legalisation in urban Alberta [Q=7] | Retrospective data of urban Alberta (Calgary and Edmonton) from National Ambulatory Care Reporting System and HealthLink and Alberta Poison and Drug Information Service public telehealth call databases; N=11,770 pre-legalisation and N=2962 post-legalisation visits, 2013-2018 and 2019 | Cannabis-related presentations | Cannabis-related ED visits | IRR=1.45 [1.39, 1.51], +43.48 [26.52, 60.43] visits per month | + | Post-legalisation, there was an increase in cannabis-related ED visits, hyperemesis, and poison centre calls but with fewer hospital admissions |  |
|  |  |  | Poison service calls | IRR=1.87 [1.55, 2.37], +4.02 [0.11, 7.94] visits per month | + |  |  |
|  |  |  | Cannabinoid hyperemesis | RR=1.23 [1.10, 1.36] | + |  |  |
|  |  |  | Unintentional ingestion | RR=1.48 [1.34, 1.62] | + |  |  |
|  |  |  | Hospital admissions | RR=0.88 [0.80, 0.96] | - |  |  |
| Yeung (2021): To compare cannabis-related ED pediatric visits in Alberta after legalization [Q=7] | Retrospective National Ambulatory Care Reporting System for urban Alberta cannabis-related ED visits, N=1920 pre, N=602 post, aged <18 years; Oct 2013 to Feb 2020 | Trends in pediatric cannabis-related ED presentation | Unintentional ingestion, 0-11 yrs | RR=1.24 [1.05, 1.47] | + | Post legalization was associated with increased cannabis-related ED presentations for unintentional ingestion in children and older adolescents, and hyperemesis in older adolescents; no significant differences were observed among younger adolescents. |  |
|  |  |  | Unintentional ingestion, 12-14 yrs | RR=1.00 [0.67, 1.51] | ns |  |  |
|  |  |  | Unintentional ingestion, 15-17 yrs | RR=1.48 [1.21, 1.81] | + |  |  |
|  |  |  | Hyperemesis, 0-11 yrs | -- | -- |  |  |
|  |  |  | Hyperemesis, 12-14 yrs | RR=1.51 [0.52, 4.43) | ns |  |  |
|  |  |  | Hyperemesis, 15-17 yrs | RR=1.64 [1.13, 2.37] | + |  |  |
| **Hospital presentations** | | | | | | | |
| Callaghan (2022): To compare emergency department presentations for cannabis-induced psychosis and schizophrenia after cannabis legalization [Q=7] | Emergency department presentations across Alberta and Ontario; 2015-2019 | Weekly ED presentation counts in Seasonal Autoregressive Integrated Moving Average (SARIMA) models | Cannabis-induced psychosis | +0.34 [-4.1, 4.8], p=0.88 | ns | The implementation of cannabis legalization was not associated with a significant change in ED presentations for cannabis-induced psychosis or schizophrenia. |  |
|  |  |  | Cannabis-induced schizophrenia | +24.34 [-18.3, 67.0] p=0.26 | ns |  |  |
| Jordan (2022): To compare cannabinoids in post-mortem blood samples before and after recreational cannabis legalization [Q=7] | Retrospective chart review of all adult Coroner's cases with toxicology analysis, N=3060, New Brunswick; 2014-2020 | Cannabinoid-positive blood samples after legalization | Cannabis present post-mortem, overall | pre=17.1% post=20.6%; OR=0.04 [0.00-0.08], p=0.028 | + | Cannabis use had increased after legalization in New Brunswick, particularly within young adults and those who died by suicide or accidental means. |  |
|  |  |  | Cannabis present in accidental deaths | pre=25.8% post=36.1%; OR=0.10 [0.04-0.17] | + |  |  |
|  |  |  | Cannabis present in suicide deaths | pre=18.1%  post=30.7%; OR=0.13 [0.06-0.22] | + |  |  |
| Kim (2022): To examined trends in hospitalisations in Ontario for cannabis-related diagnoses before legalisation and during two phases after legalisation [Q=6] | Ontario Health Insurance Plan eligible adults; n =12,079,699; age 18+; pre=2015-17 Oct 2018; Phase 1=18 Oct 2018-17 Mar 2020; Phase 2=18 Mar 2020-17 May 2021 | Difference in cannabis-related hospitalisations (per 100k): Pre-legalisation to Phase 1 to Phase 2; Phase 1 (October 2018-March 2020), flower and herb sales online and limited private retail storefronts;  Phase 2 (Mar 2020-May 2021), increased storefronts and availability of edibles (Mar 2020-May 2021) | Cannabis-related hospitalisations: Women 18-24 | pre=6.05  P1=7.77  P2=11.11; immediate change P1=38% p=0.012, P2=15.3% p=0.267 | + then ns | Before legalization, there were significant month-to-month decreases in women 18–24 and men aged 18–24 but the incidence rate ratio increased by 1.5% per month for men over 25 years. Phase 1 was associated with a significant immediate increase and month-to-month increase in hospitalization in all age and gender subgroups, with greater increases in men than women. Phase 2 was not associated with immediate significant increases in hospitalization in adults aged 18–24 or 25 years plus and the month-to-month trend in each subgroup did not significantly differ. |  |
|  |  |  | Men 18-24 | pre=5.32  P1=11.59  P2=15.18; immediate change P1=77.8 p<0.001, P2=-13.8% p=0.275 |  |  |  |
|  |  |  | Women >=25 | pre=0.67  P1=1.49  P2=2.11; immediate change P1=52.9% p=0.001, P2=15.1 p=0.219 |  |  |  |
|  |  |  | Men >=25 | pre=0.97  P1=3.19  P2=4.16; immediate change P1=73.6% p<0.001, P2=-11.2% p=0.243 |  |  |  |
| Maloney-Hall (2020): To examine pre-legalisation trends in cannabis-related hospitalizations for mental and behavioural disorders [Q=7] | Inpatient separations data from the Canadian Institute for Health Information, N=8819 cannabis-related mental disorder separations; 2006-2015 (2-yrs post liberal medical cannabis laws) | Inpatient separations with a primary diagnosis of mental and behavioural disorder | Cannabis-related mental disorders | 2.11 in 2006 vs 5.18 in 2015, per 100k | + | Prior to cannabis legalization, comparisons over time showed that the rates of hospitalizations for cannabis-related mental or behavioural disorders increased from 2006-2015, highest proportions in young males aged 15-24 for psychotic disorders |  |
|  |  |  | Cannabis-related psychotic disorder | 0.80 in 2006 vs 2.49 in 2015, per 100k | + |  |  |
| Vignault (2021): To examine the impact of cannabis legalization on the prevalence of cannabis use disorder and psychotic disorder [Q=7] | Retrospective observational study of patients aged 12+ who visited a psychiatrist in the emergency unit of the Centre hospitalier universitaire de Sherbrooke (CHUS) in Quebec, n=1247, 2-years before vs 5-months after legalization of cannabis for recreational use | Prevalence of cannabis use disorder and psychotic disorder in psychiatric presentations | Reported use of cannabis | pre=28%  post=37% | + | Cannabis use disorder presentations increased after legalisation, psychotic diagnosis had not, and personality disorder had. This study covered a short period after legalisation and the data may be affected by ascertainment bias. |  |
|  |  |  | Cannabis use disorder | pre=18%  post=24% | + |  |  |
|  |  |  | Psychotic diagnoses | pre=27%  post=29% | ns |  |  |
|  |  |  | Personality disorder | pre=40%  post=45% | + |  |  |
| *Note.* Canada legalized cannabis use for medical purposes in 1999, and for recreational use in Oct 2018; *Effect size: B=beta; β=standardized beta; D=mean differences; FU=follow-up; IRR=incidence rate ratio; M=mean; PR=prevalence ratio; n/a= not applicable; OR=odds ratio; Impact key: + increased, - decreased, ns: no significant differences. Q=Quality out of 8;* *Joanna Briggs Institute (JBI) Critical Appraisal Tool.* | | | | | | | |
